# Ten open questions in migraine prophylaxis with monoclonal antibodies blocking the calcitonin-gene related peptide pathway: a narrative review

**DOI:** 10.1186/s10194-023-01637-7

**Published:** 2023-08-01

**Authors:** Jean Schoenen, Annelies Van Dycke, Jan Versijpt, Koen Paemeleire

**Affiliations:** 1 Headache Research Unit, Department of Neurology‑Citadelle Hospital, University of Liège, Boulevard du 12 ème de Ligne 1, Liège, 4000 Belgium; 2 Department of Neurology, General Hospital Sint-Jan Bruges, Ruddershove 10, Bruges, 8000 Belgium; 3 Department of Neurology, Vrije Universiteit Brussel (VUB), Universitair Ziekenhuis Brussel (UZ Brussel), Laarbeeklaan 101, Brussels, 1090 Belgium; 4 Department of Neurology, Ghent University Hospital, Corneel Heymanslaan 10, Ghent, 9000 Belgium

**Keywords:** CGRP, Monoclonal antibodies, Physiological effects, Clinical practice, Contraindications, Migraine

## Abstract

The monoclonal antibodies (mAbs) blocking the calcitonin-gene related peptide (CGRP) pathway, collectively called here “anti-CGRP/rec mAbs”, have dramatically improved preventive migraine treatment. Although their efficacy and tolerability were proven in a number of randomized controlled trials (RCTs) and, maybe even more convincingly, in real world settings, a number of open questions remain. In this narrative review, we will analyze published data allowing insight in some of the uncertainties related to the use of anti-CGRP/rec mAbs in clinical practice: their differential efficacy in migraine subtypes, outcome predictors, switching between molecules, use in children and adolescents, long-term treatment adherence and persistence, effect persistence after discontinuation, combined treatment with botulinum toxin or gepants, added-value and cost effectiveness, effectiveness in other headache types, and potential contraindications based on known physiological effects of CGRP. While recent studies have already provided hints for some of these questions, many of them will not find reliable and definitive answers before larger studies, registries or dedicated RCTs are available.

## Introduction

The monoclonal antibodies (mAbs) blocking calcitonin-gene related peptide (CGRP) (eptinezumab, fremanezumab, galcanezumab) or its receptor (erenumab), collectively called here “anti-CGRP/rec mAbs” have proven their efficacy and safety in multiple large randomized controlled trials (RCT). They are now universally recommended for the preventive treatment of both episodic (EM) and chronic migraine (CM), though not as first line therapies chiefly for pharmaco-economic reasons [1]. A number of real-world studies have confirmed their effectiveness, but also unraveled some overlooked adverse effects and provided useful information for clinical practice [2–4]. Despite the large body of scientific data, there remains a number of open questions concerning their effect in subtypes of migraine, predictors of (in)efficacy, long term management strategies, combination with other treatments, cost-effectiveness, effect in other headache types and, given the known myriad of physiological actions of CGRP, the potential adverse effects and possible contraindications due to its blockade in the long term. In the following sections, we will address the 10 most pertinent questions in a systematic way.

## Is their efficacy identical in all migraine subtypes?

### Migraine with aura

The physiological effects of CGRP, in particular on vessels [5, 6] and oxidative stress (reviews in 7,8) could play a role in the pathophysiology of migraine auras. In theory the large molecular weight of anti-CGRP/rec mAbs prevents their penetration through the blood-brain barrier (BBB) and in rat, even after opening the BBB, fremanezumab did not inhibit cortical spreading depression (CSD) [9] or CSD-induced arterial dilatation and plasma protein extravasation [10]. A study using transcranial Doppler sonography, CGRP infusion induced a greater vasodilatatory reponse in the posterior circulation in migraine with aura than in migraine without aura patients; the authors suggest that this could favour CSD assuming that CGRP would dilate chiefly the proximal arteriolar segments while the distal segments would constrict due to local hyocapnia to maintain a constant cerebral blood flow, a hypothesis that needs to be proven [11].

In a post-hoc secondary analysis of 4 RCTs with erenumab, no significant difference in monthly migraine day (MMD) reductions was found between patients with or without a history of migraine with aura, although at the 140 mg dose the reduction and gain over placebo was lower in EM patients with a history of aura. As expected, erenumab had no effect on monthly number of aura days in the CM group where this outcome was assessed [12].

In a recently published observational, open-label cohort study of 46 patients with high frequency episodic migraine (HFEM) or CM treated with galcanezumab for 3 months, the incidence of headache after the occurrence of visual aura was reduced by 50% in super-responders ($\ge$ 70% reduction of migraine days), by 0% in super non-responders ($\le$30% reduction of migraine days), while there was similarly a greater decrease in headache incidence after prodromal symptoms in super-responders [13].

During real-world treatment with anti-CGRP/rec mAbs, contradictory changes of aura attacks were reported, ranging from aura frequency decrease in 35% of patients [14] to no change in most patients [15] or de novo occurrence of auras in a handful of patients [15, 16] or worsening in one patient [17].

Further randomized controlled studies are needed to determine if the anti-CGRP/rec mAbs are able to modify the incidence of aura attacks, or if their effect is limited only to a decreased incidence of headache following an aura, as suggested by Ashina et al’s study [13]. It is reassuring, however, that there is up to now no indication from real-world experience for a deleterious effect of the anti-CGRP/rec mAbs on severity of migraine auras.

### Chronic migraine

According to most RCTs and real-world studies, the efficacy of anti CGRP/rec mAbs is not significantly different between EM and CM [3, 18–20] including the most difficult-to-treat patients with numerous previous preventive treatment failures [21, 22]. However, in some real-world trials non-responders are more numerous in CM than in EM [15, 23]. This could be due to the fact that CM is far from being a homogeneous condition. It comprises at least two major subgroups of patients, tentatively identified in the Appendix of the 3rd edition International Classification of Headache Disorders (ICHD-3) [24], those with *pain-free periods* (code A1.3.1) and those with *continuous pain* (code A1.3.2) defined as headache not interrupted by pain-free periods of > 3 h on $\ge$5 days/month. With a few exceptions [25, 26], CM patients with continuous pain have been excluded from most RCTs with anti-CGRP mAbs [27]. In real-world studies continuous or daily headache is a negative predictor of treatment response [15, 28, 29] (Table 1), which might explain the lower response rates found in CM cohorts with a combination of both subgroups of patients [15, 23].

**Table 1:** Possible predictors of (in) efficacy

*Clinical features*	*Anti-CGRP/rec monoclonal antibody*
**POSITIVE PREDICTORS** (*post-hoc analysis)*	
Unilateral headache [52, 53]	erenumab galcanezumab
Presence of cranial autonomic symptoms [54]	erenumab, fremanezumab, galcanezumab
Less severe disability [34, 55]	erenumab
Higher baseline migraine frequency [28, 52, 55]	erenumab
Good response to triptans [23, 53, 56]	erenumab galcanezumab
Vomiting, all typical migraine features, good response to triptans more frequent in super-responders ($\ge$ 75%) [23]	erenumab, fremanezumab, galcanezumab
Absence of other headache types [28, 56]	erenumab
Younger age [36]	fremanezumab
Higher susceptibility to CGRP-triggered attacks [58]	erenumab
Higher pre-treatment salivary CGRP [59]	erenumab
**Treatment-induced changes associated with good outcome**	
50% reduction of MIDAS or monthly migraine days at 3 months [57]	erenumab
Increased thresholds of biceps femoris withdrawal reflex at 3 months [60]	erenumab
Lower serum CGRP after 4 weeks (but not pre-treatment) [61]	erenumab
Less iron accumulation in PAG and anterior cingulate cortex at 8 weeks post-injection [62]	erenumab
**NEGATIVE PREDICTORS** (*post-hoc analysis)*	
Chronic migraine [15, 23]	erenumab, fremanezumab, galcanezumab
Chronic migraine with continuous pain [15, 17, 28, 29, 49]	erenumab
Medication-overuse headache [23, 34]	erenumab, fremanezumab, galcanezumab
Comorbid depression [23]	erenumab, fremanezumab, galcanezumab
Multiple previous preventive failures [15, 28, 33, 34]	erenumab
Higher baseline migraine frequency [28, 35]	erenumab
Interictal cephalic allodynia [51]	galcanezumab

### Medication overuse headache

Excessive use of acute medications is, together with high attack frequency and depression [30], the most important risk factor for migraine chronification and associated with so-called *medication overuse headache* (MOH) (ICHD-3 8.2). In RCTs where CM patients with medication overuse were subanalyzed, efficacy of all 4 anti-CGRP/rec mAbs was comparable to that found in patients without such overuse [31, 32]. This was also the case in most real-world studies [15], although in some studies outcome was less favorable in patients with MOH [33–36]. In a retrospective cohort study comparing super-responders ($\ge$75% reduction in monthly headache days-MHDs) and non-responders ($\le$25% reduction) after 3 months of treatment with erenumab, galcanezumab or fremanezumab, medication overuse was more frequent in the latter (58% vs. 28%) [23] (Table 1). In a prospective, randomized, open-label study of patients with CM and MOH the addition of erenumab, fremanezumab or galcanezumab to overused medications withdrawal resulted in a significantly higher reduction of headache days and symptomatic medication intake [37]. In a prospective study of erenumab and galcanezumab, efficacy was similar in patients who were detoxified in-hospital prior to the start of treatment and in those who were not [38].

Efficacy in MOH patients was also reported for onabotulinumtoxinA (BoNT-A) in a subanalysis of the pooled PREEMPT trials [39], but in a meta-analysis [40] this was true for reduction in monthly migraine days (MMDs) but not for response rate and the effect size of anti-CGRP/rec mAbs was greater, and drop-out rates were lower than those of BoNT-A. In pooled RCTs [41] topiramate was effective in CM with MOH, but numerically more effective in CM without MOH. Giri et al. [40] concluded from their systematic review that “there is currently insufficient evidence to determine the impact of topiramate in CM with MOH”.

There is thus, on the one hand, clinical evidence that anti-CGRP/rec mAbs are effective in CM with MOH. On the other hand, however, 50% of such patents improve, i.e. may reverse to EM, 2 months after simple withdrawal of the overconsumed drug [42] and several studies showed that preventive therapy is the most effective when it is associated from the start on with withdrawal [43–45]. From a pharmacoeconomic point of view, it might thus be appropriate to withdraw MOH patients from overuse, not only at the start of any prophylactic migraine treatment, but also before considering 3rd or 4th line therapies with expensive drugs like anti-CGRP/rec mAbs or BoNT-A, which is currently not requested by most reimbursement policies.

## Are there any outcome predictors?

Given that the anti-CGRP/rec mAbs are inefficient in a proportion of patients and a costly therapy, it would be useful for the practitioner (and the patient) to be able to predict who is likely to be a responder. A number of predictors of effect have been retrospectively identified in RCTs or real-world studies as well as post-treatment changes correlating with successful outcome (Table 1), but their positive or negative predictive value is mostly not high and hence of low or uncertain usefulness in individual patients.

In most trials, previous failures of preventive treatments do not prevent anti-CGRP/rec mAbs from being effective [2, 22]. An inverse relation between outcome and number of prior treatment failures was nonetheless reported in several real-world studies [15, 33, 34]. Moreover, although the LIBERTY trial [46] is taken as evidence that erenumab is effective even after 2–4 previous treatment failures in EM, the reported 50% responder rate of 30% is clearly lower than that of the pivotal RCTs for EM (50%) [47] or even CM (41%) [48].

As mentioned above, CM patients with continuous pain (ICHD-3 A1.3.2) may be poor responders to anti-CGRP/rec mAbs and probably to most treatments. During a compassionate use program of erenumab [15], very low 50% and 30% responder rates, of respectively 13% and 37%, were found in such a cohort of patients, contrasting with 58% and 76% in CM patients with pain-free periods (ICHD-3 A1.3.1). Similarly, other real-world studies found a poor outcome with erenumab in such patients [17, 28, 29] and no improvement by switching from erenumab to a ligand-blocking mAb in patients with initially daily headache [49]. Additional pathophysiologic and therapeutic studies are clearly needed in CM patients with continuous pain, the more so that they also respond poorly to neuromodulation treatments [50]. Psychiatric comorbidity may be a culprit in these patients according to one study where concomitant depression was more frequent in non-responders (65%) than in super-responders (28%) [23].

Higher baseline migraine frequency [28, 35] and interictal cephalic allodynia [51] have also been reported as poor outcome correlates.

Although with some discrepancies, positive *post-hoc* predictors of treatment success and treatment-induced changes associated with good outcome were also identified in real-world studies, most of them with erenumab (Table 1): headache unilaterality [52, 53], cranial autonomic attack symptoms [54], higher baseline migraine frequency [51, 55], less severe disability at baseline [34, 55], absence of other primary headaches [28, 56], good response to triptans [23, 53, 55], typical migraine features and vomiting [23], young age [36], and a $\ge$ 50% response after 3 months of treatment [57]. In experimental studies, responders to erenumab had higher susceptibility to attack induction by the intravenous administration of CGRP [58], higher pre-treatment salivary CGRP levels [59], increased thresholds of the biceps femoris withdrawal reflex at 3 months [60], lower serum CGRP levels at 4 weeks [61], and less iron accumulation in the periaqueductal gray and anterior cingulate cortex at 8 weeks post-injection [62].

## Is switching between anti-CGRP/rec mAbs useful?

Various recent network meta-analyses of RCTs have confirmed similar efficacy and tolerability profiles for erenumab, fremanezumab, galcanezumab and eptinezumab [19, 63, 64]. This was confirmed in one prospective, observational cohort study that found no evidence suggesting superiority of one antibody over the other [65], but a recent study suggested that the ligand-blocking mAbs might be modestly but significantly more effective than erenumab, the receptor-blocking mAb [66]. Individual differences in treatment response may exist between anti-CGRP/rec mAbs. Switching between them may thus be appropriate in selected patients. Its therapeutic value is up to now only suggested by a handful of case series and seems unpredictable. Ziegeler & May [67] reported on 3 patients (2 CM, 1 EM) not responding to erenumab who were all three significantly improved by galcanezumab. In another case series, 3 out of 7 CM patients benefited from a switch between anti-CGRP/rec mAbs [68]. Switching to a second mAb in 14 patients with CM was followed by a persistent amelioration in 9 of them [69]. In a larger retrospective diary review [49], 25 patients (*n* = 22 CM) who had < 30% reduction of MHDs after 3 treatment cycles to erenumab, switched to galcanezumab (*n* = 12) or fremanezumab (*n* = 13). Three months after switching 3 out of 25 were $\ge$50% responders while 8 out of 25 had a 30% response; patients with daily headache had no response. In another retrospective study of 22 patients (19 CM) not responding to a first anti-CGRP/rec mAb, switching to a second mAb produced a 75% response in 1 patient, a $\ge$ 50% response in 6 patients and a $\ge$ 30% response in 3 patients; no difference was found between switching against a ligand- or a receptor-blocking mAb nor, by contrast with the previous study, between daily versus non-daily headache patients [70]. Data on switching to eptinezumab are not yet available.

To summarize, as stated in the updated EHF recommendations [1], there is at present insufficient evidence on the potential benefits of antibody switching, although a minority of patients may benefit from it. In theory, it seems rational to switch between different classes of antibodies, i.e. from erenumab, the CGRP receptor blocker, to a mAb blocking the ligand, or vice versa. It remains to be demonstrated whether this is the most effective strategy, but preliminary data suggest that it may not be relevant [70]. In clinical practice very few patients may benefit from a switch to a 3rd anti-CGRP/rec mAb. If switching is considered after 12 weeks of treatment, a timepoint at which most patients will have responded or not [57], one has to take into account the recent results from an Italian registry showing that 146 out of 265 non-responders (55.1%) to an anti-CGRP/rec mAb at 12 weeks have nevertheless a $\ge$ 50% response after 24 weeks [71].

## Can anti-CGRP/rec mAbs be used in adolescents and children?

In an opinion paper, the members of the Pediatric and Adolescent Headache special interest group of the American Headache Society (AHS) caution against an unrestricted use of anti-CGRP/rec mAbs in pediatric and adolescent migraine patients until the data from the ongoing RCTs in these age groups are available [72]. Their use may nonetheless be considered in appropriate cases refractory to at least 2 other preventive drugs taken for 2–3 months and non-drug treatments, with the lowest effective dose and the shortest possible treatment duration, given the physiological role of CGRP in bone formation [73]. Regarding the latter, however, available data are not totally concordant. While in animal experiments, CGRP was shown to stimulate osteoblast differentiation and to inhibit osteoclast formation [74], a recent prospective cohort study of 45 CM patients treated for 3 months with a ligand-targeting anti-CGRP mAb (91.1% galcanezumab, 8.9% fremanezumab) found a significant increase in a serum marker of bone formation, but no change of a bone resorption marker [75]. Close monitoring of pubertal status, bone health, linear growth and BMI are nevertheless recommended in young migraineurs treated with CGRP pathway blocking drugs. Spzerka et al. [72] mention as contraindications: disturbed blood-brain barrier (recent meningitis or neurosurgery), severe cardiovascular disease or stroke, as well as pregnancy and breast feeding, which are also of concern in adults (see Table 3).

The cost considerations of the AHS consensus were challenged by Charles and Turner [76] who argued that “cost considerations is not a goal of treatment. Our task is to treat our patients with effective therapies that are safe and without adverse effects, not cheap drugs first”, a statement that may be applicable in a system with chiefly private health insurance, like in the USA, but not in a public health insurance system with a budgetary envelope, like in most European countries. Meanwhile, in a retrospective study of 112 adolescents (mean age:15.9 yrs) with refractory chronic headache disorders (83.9% CM), treated with an anti-CGRP/rec mAb (86.6% erenumab), 30% had a “significant benefit” defined as $\ge$ one third reduction in headache frequency, intensity or duration for at least 1 month, and 40% had “some benefit”. There was a modest reduction of -2.0 MHDs. Tolerance was excellent with only 4.5% of subjects discontinuing treatment because of adverse effects that were similar to those reported in adults [77].

Until the results of ongoing pediatric RCTs [78] are published, treatment with an anti-CGRP/rec mAb in migraine patients below age 18 cannot be advocated without the abovementioned precautions and is not yet reimbursed in several countries.

## What about long-term adherence and persistence?

Adherence and persistence to treatment are major problems with the classical oral preventive migraine drugs, especially in CM. In a retrospective US claims analysis of 8707 CM patients, persistence to the initial preventive medication was 25% at 6 months and 14% at 12 months; in patients who switched to another preventative persistence was between 10 and 13% [79]. Adherence ranged between 26% and 29%, being lowest for amitriptyline, nortriptyline, gabapentin and divalproex [80].

In RCTs of anti-CGRP/rec mAbs discontinuation of treatment during the double-blind phase was exceptional. For instance, in EM the number of patients that need to be treated with erenumab 140 mg [81] to experience an adverse event leading to treatment discontinuation is 319 while the corresponding figure for topiramate 100 mg is 7 [82]. In an open-label 5-year extension phase with erenumab [83], 34.5% (132/383) of EM patients discontinued erenumab 70 mg within 2 years, most of them because they requested so, were not ameliorated or had adverse effects. When the erenumab dose was increased to 140 mg/month after 2 years, 36 of 250 (14%) patients discontinued treatment for the same reasons, but seemingly only 138 patients (55%) were still treated at the 5-year term. In patients who remained in the extension phase for 5 years, treatment efficacy (50% responder rate of 71%, non-adjusted for drop-outs), tolerance (exposure-adjusted adverse event rate: 123/100 patient years) and safety (no new signals) were stable.

In real-world studies of erenumab, drop-out rates for lack of efficacy ranged from 1.4 to 1.9% after 12 weeks [52, 84] to 40% after 6 months [17, 85]. After 1 year of follow-up, adherence to erenumab was around 70% in two surveys [15, 86]. Interestingly, in one of these studies [86] 59.3% of patients escalated from the 70 mg to the 140 mg of erenumab over 1 year, while only 4.4% deescalated from 140 to 70 mg. Among 160 resistant CM patients treated with erenumab 47% still had a $\ge$30% response after 2 years in one study [87] and 54.8% continued treatment after 17–30 months in another one [88]. In a Danish long-term, observational study of 300 CM patients treated with erenumab, 40% provided data at 52 weeks and a sustained ≥ 30% reduction in MMDs at all assessment timepoints throughout the 52-week treatment period was achieved by 34% of patients [89]. It is worth mentioning that in the multicenter European ESTEEM study, even patients with a good relative response to erenumab had a residual clinically relevant burden: after 12 weeks of treatment among the 32.6% patients with a 50% MMD reduction versus baseline (396 out of 1215 patients) 62% still had 4–7 MMDs and 23.7% even had 8–14 MMDs [90].

There is thus little doubt that treatment persistence and adherence is better with the anti-CGRP/rec mAbs than with previous migraine preventatives. Nonetheless, even in good responders a significant burden may persist because of residual migraine attacks and within 2 years over 30–40% of patients are likely to discontinue treatment because of inefficacy or, less frequently, due to intolerance.

## How long does the effect persist after treatment discontinuation?

Numerous studies have assessed the effect duration of anti-CGRP/rec mAbs after discontinuation. Overall, they indicate that the effect persists for only a few weeks or months and does hardly outlast their pharmacological action. Cessation of treatment after 6 months in the pivotal galcanezumab RCTs Evolve-1 and Evolve-2 showed that more than 25% of patients lost their 50% response after 1 month, 45% after 2 months and 60% after 4 months, although on average the number of MMDs remained below baseline levels [91]. All real-world studies showed a waning of the effect 2–3 months after treatment discontinuation, some as soon as 1–4 weeks after completion of a 1-year treatment [54, 92–94]. Out of 24 patients who interrupted erenumab for at least 3 months and had $\ge$ 8 MMD in the 3rd month, 14 patients (58%) had already $\ge$ 8 MMD in the 2nd month [92]. In the hitherto largest longitudinal study of 154 patients treated with erenumab or galcanezumab, the 50% responder rate dropped 3 months after treatment cessation from 73 to 27% in HFEM (*n* = 47) and from 60 to 35% in CM (*n* = 107) [95]. Size and onset of the clinical deterioration after treatment discontinuation were not significantly different between these two anti-CGRP/rec mAbs. However, in a study focusing on the post-treatment changes of headache impact and health-related quality of life, a slightly more rapid deterioration was found in patients treated with erenumab than in those treated with galcanezumab or fremanezumab, which might be due to the longer half-life or a more pronounced efficacy of the latter [96]. In 44 chronic migraine patients treated for 12 months with erenumab or galcanezumab, one quarter showed a sustained benefit during a 3-month discontinuation period and did not need retreatment; the only post-hoc positive predictor of sustained benefit was lower pre-treatment disability as indexed by lower Migraine Disability Assessment (MIDAS) and Headache Impact Test 6 (HIT) scores [97], Most patients restart treatment after a planned drug holiday, but in the real-world study by Raffaelli et al. [98] 11 of 39 patients (28.2%) did not achieve a $\ge$ 30% response to the same mAb after resumption of erenumab (*n* = 5) or galcanezumab/fremanezumab (*n* = 6). This finding needs to be replicated and, if confirmed, the reason for a poorer response to a 2nd treatment period remains to be determined.

Early recurrence of migraine headaches after anti-CGRP/rec mAb discontinuation is at odds with topiramate. After 6 months of treatment with the latter the therapeutic benefit was maintained up to 6 months after cessation in a placebo group although MMDs increased 1.09 days more than in patients who stayed on topiramate [99]. However, similar blinded trials evaluating the efficacy after treatment discontinuation are at present not available for anti-CGRP/rec mAbs. The available evidence that little of their therapeutic effect outlasts their pharmacological effect favors the hypothesis that anti-CGRP/rec mAbs, contrary to other preventive treatments, act chiefly as long-lasting acute therapies at the level of the trigeminovascular system [2]. Whether this could be related to the persistence of “phantom attacks” without headache during treatment [2, 100] remains to be determined. It also remains to be confirmed if anti-CGRP/rec mAbs reduce interictal burden, independently of the reduction in attack frequency. This is suggested by a significant decrease of the Migraine Interictal Burden Scale after 3 and 6 months of galcanezumab treatment in EM and CM patients with 2–4 previous preventive treatment failures [101], but the decrease in interictal burden could be in part due to the decreased likelihood of an attack occurrence.

Taken together, published real-world data indicate that most patients worsen significantly as soon as the 2nd month of anti-CGRP/rec mAb treatment discontinuation. Although it seems reasonable to limit treatments to patients who benefit from them and to evaluate periodically the sustained need for migraine prophylaxis, a prescheduled treatment holiday and a fixed (3-month) duration of treatment interruption, as mandatory for reimbursement purposes in several countries, may not be adequate.

## Can anti-CGRP/rec mAbs be combined with botulinum toxin and gepants?

Given that BoNT-A prevents the activation of nociceptive C fibers while anti-CGRP/rec mAbs mainly blocks A$\delta$ fibers, there may be a physiological rationale for an association of both in CM treatment [102]. As a matter of fact, several studies have shown the benefit of adding an anti-CGRP/rec mAb in CM patients not responding adequately to BoNT-A, which produced a clinically meaningful improvement in $\pm$40% of patients [103–107]. In 19 CM patients who had less than 30% reduction in MHDs with BoNT-A (*n* = 19) or fremanezumab (*n* = 17) or erenumab (*n* = 2) as monotherapies, the combination of both BoNT-A and an anti-CHRP/rec mAb resulted in $\ge$50% of MHDs in 14 patients [108]. The combination does not induce additional adverse effects or new safety signals. In a real-world study of 155 CM patients, however, in which erenumab and galcanezumab were found efficient after complete or partial failure on previous BoNT-A, dual therapy in 12 patients had no additional benefit [109]. Accordingly, a retrospective chart review comparing CM patients treated with erenumab alone (*n* = 70) or as an add-on to BoNTA (*n* = 73) found that the reduction in MHDs was less with the dual therapy (-4.7) than with erenumab (-8.2) and the probability of achieving a $\ge$ 50% reduction in MHDs lower with the dual therapy (odds ratio: 0.57) [110].

The novel small molecule CGRP receptor antagonists (gepants) can safely be added as attack treatment during preventive anti-CGRP/rec mAbs treatment [111, 112] and co-administration in migraine patients did not influence the pharmacokinetics or safety of ubrogepant [113]. The same good tolerance remains to be proven for the long-term combination of a CGRP/rec mAb with a gepant administered as preventive therapy, as well as the possible benefit of such combination.

## What is their added-value and cost-effectiveness?

The only available RCT (HER-MES) comparing directly an anti-CGRP receptor mAb, erenumab, and a classical migraine preventive, topiramate, for HFEM showed a clear therapeutic advantage of the former over the latter: 60% of patients receiving erenumab had a $\ge$50% MMD reduction after 6 months compared to 43% in the topiramate group while 10% of patients discontinued erenumab due to adverse effects compared to 39% with topiramate [114, 115]. Indirect comparisons of RCTs performed with classical preventive drugs or anti-CGRP/rec mAbs confirm that the latter have an unprecedented favorable ratio between efficacy and tolerance [116] and thus a markedly greater likelihood to help than to harm compared to propranolol, topiramate or BoNT-A [82]. In an adjusted indirect treatment comparison meta-analysis of 10 trials in CM, galcanezumab and fremanezumab reduced migraine days more than BoNT-A at week 12, whereas the reduction in headache days was similar as were adverse event rates [117]. A meta-analysis in CM with MOH concluded that both BoNT-A and anti-CGRP/rec mAbs are beneficial in reducing MMDs, but the effect size for the latter is greater and the drop-out rate lower [40].

There is strong evidence from studies using various patient-reported outcome measures that migraine treatment with anti-CGRP/rec mAbs improves quality of life, disability and work productivity, and reduces health resource utilization as well as the expense for acute medications [2, 118]. Although these studies suggest that CGRP/rec mAbs are also cost-effective, their high pricing, incomplete effectiveness and assumptions that less expensive and equally well-tolerated treatment alternatives might be as effective [119] underscore the need for pharmaco-economic analyses. In a pharmaco-economic study performed in Greece [120] incremental cost-effectiveness ratios (ICERs) for the treatment of CM with erenumab versus BoNT-A were €218,870 (indirect costs included) per quality-adjusted life year (QALY) gained and €620 per migraine day avoided. For the erenumab ICER to fall below the cost-effectiveness threshold equal to three times the local gross domestic product per capita (€49,000), the price of erenumab would have to be no more than €192 per dose (societal perspective), which is substantially lower than the present prices in most countries, but needs of course to be adjusted to each country’s gross domestic product per capita. A systematic review of 16 economic evaluations of pharmacological treatments for adults with CM [121] concluded that erenumab, fremanezumab and galcanezumab, were associated with ICERs ranging between 81,080 € and 218,870 €, above the most common willingness-to-pay thresholds (WTPs), compared to 17,720€-19,572€ for BoNT-A. The anti-CGRP/rec mAbs, however, were cost-effective within the commonly used WTPs among the patient population for whom previous preventive treatments, including BoNT-A, had failed.

Taken together, the added-value of anti-CGRP/rec mAbs is most obvious when both their effect size and adverse effect profile are compared to those of classical preventive drugs. Although in a real-world situation they clearly reduce acute medication use, disability and utilization of health resources, proving their cost-effectiveness in pharmaco-economic studies has been difficult and most convincing in the most disabled patients, predominantly when indirect costs are included. These difficulties are in part due to the high pricing of anti-CGRP/rec mAbs.

## Are they effective in other headache types?

Anti-CGRP/rec mAbs have been used to treat various other disorders than migraine in some RCTs but most often in retro- or prospective case series (Table 2).

**Table 2:** Possible efficacy in other disorders

*Disorder*	*Author and study type*	*Anti-CGRP mAb and effect*
Episodic Cluster Headache	Goadsby et al. 2019 [123] (**RCT**) Plato et al. 2021 [125] (**RCT**) Cited by Carmine Belin et al. 2020 [122] (**RCT**)	galcanezumab *> placebo* fremanezumab *(trial discontinued for futility)*
Chronic Cluster Headache	Dodick et al. 2020 [126] (**RCT**) Ruscheweyh et al. 2020 [127], Silvestro et al. 2020 [128] (**case series**)	galcanezumab *= placebo* galcanezumab - *possibly effective*
Persistent post-traumatic headache	Ashina et al. 2020 [130] (**prospective, open label**) McVige et al. 2022 [131] (**retrospective chart review**) Spierings et al. 2023 (abstract) [132] (**RCT**)	erenumab - *marginally effective* erenumab, fremanezumab or galcanezumab – *possibly effective* fremanezumab *= placebo*
Vestibular migraine	Hoskin & Fife 2022 [134] (**case series**) Russo et al. 2023 [135] (**prospective, open label**)	erenumab, fremanezumab, galcanezumab - *probably effective*
Idiopathic intracranial hypertension with persistent headache	Yiangou et al. 2021 [136] (**prospective, open label**) Frerichs et al. 2022 (abstract) [137] (**retrospective chart review)**)	erenumab - *possibly effective* erenumab, fremanezumab, galcanezumab - *ineffective*
Trigeminal neuralgia	Parascandolo et al. 2022 [136] (**case series**) Schott Andersen et al. 2022 (**RCT**) [137]	erenumab – *possibly effective* erenumab *= placebo*
Mitochondriopathy with chronic migraine with aura	Naegel et al. 2021 [140], Kaltseis et al. 2022 [141] (**case reports**)	erenumab or galcanezumab - *possibly effective*
Nummular headache	Lopez-Bravo et al. 2022 [142] (**case report**)	galcanezumab - *possibly effective*
Neuropathic pain comorbid with chronic migraine	Kang & Govidarajan 2020 [143] (**case series**)	anti-CGRP mAb not specified – *possibly effective*

Although the ictal increase in blood levels of CGRP tends to be smaller than in migraine, there is a convincing physiological rationale for the utility of anti-CGRP/rec mAbs in *cluster headache* (CH) [122]. A RCT comparing galcanezumab (300 mg at baseline and at 1 month) and placebo in episodic CH found indeed that the mAb significantly reduced weekly frequency of attacks with a 50% reduction at week 3 in 71% of patients compared to 53% for placebo [123]. The median time-to-first occurrence of $\ge$ 50% reduction from baseline in CH attacks was 5 days for galcanezumab versus 14 days for placebo [124]. This study led to the FDA approval of galcanezumab for the preventive treatment of episodic CH. A beneficial effect of galcanezumab in episodic CH was confirmed in a smaller placebo-controlled trial [125]. Conversely, a RCT of galcanezumab in chronic CH did not achieve its primary or key secondary endpoints [126]. Erenumab, the mAb blocking the CGRP receptor, was found effective for the prevention of chronic CH attacks in small case series [127, 128], while a clinical trial of fremanezumab was discontinued after failing to meet the primary endpoint also in episodic CH [122]. A RCT of eptinezumab in episodic CH is ongoing (NCT04688775).

Overall, these studies suggest that anti-CGRP/rec mAbs are less effective in CH than in migraine, which is likely due to differences in pathophysiology but also to suboptimal trial methodologies poorly adapted to the natural history of CH, as highlighted in the recently updated IHS guidelines for clinical trials in CH [129]. They also underscore the differences in pathophysiology and response to treatments between the episodic and chronic forms of the disorder.

In a non-randomized, open-label study of 89 patients with *persistent post-traumatic headache (PTH)*, erenumab (140 mg/month) was associated with a modest improvement, reducing MHDs by 1.7 (from 24.6 at baseline to 22.9 days at 12 weeks) with 13% of patients having a $\ge$ 50% response [130]. Along the same line, a large retrospective chart review of 168 concussion patients with PTH showed that anti-CGRP/rec mAbs were associated with an average improvement of monthly headache days by -7.25 and HIT-6 scores by -4.26; headache severity and frequency, as well as overall concussion symptoms, were also improved [131]. In a phase 2 trial, not yet published *in extenso*, there was however no difference between placebo and fremanezumab in persistent PTH [132].

These discrepant results could de due to the clinical heterogeneity of PTH, where the CM phenotype could be more responsive to CGRP-blocking agents. This needs to be proven in an adequate trial, but it is suggested by a RCT showing that intravenous infusion of CGRP induced migraine-like headache in 21 of 30 participants (70%) with persistent PTH, compared with 6 of 30 participants (20%) after placebo infusion [133].

Open studies and case reports have explored the possible value of anti-CGRP/rec mAbs in other headache types. Fifteen out of 25 migraine patients treated with erenumab, galcanezumab or fremanezumab had “moderate to significant improvement” of their *vestibular migraine* symptoms in a retrospective study [134]. Such a beneficial effect with all three anti-CGRP/rec mAbs was recently confirmed in a prospective observational cohort study of 50 CM patients with vestibular migraine: 45 patients (90%) had a $\ge$ 50% reduction in vertigo frequency while 43 (86%) had a $\ge$ 50% reduction in headache frequency; mean monthly days with dizziness/vestibular symptoms decreased from 10.3 at baseline to 0.8 days after 12 months [135].

In a prospective open label study of 55 females with *idiopathic intracranial hypertension (IIH)* in ocular remission, but with persistent chronic headache, erenumab (140 mg/month in 52 patients) produced dramatic improvements with 50% responder rates for moderate/severe headaches of 62% at 3 month and 85% at 12 months [136]. By contrast, such a beneficial effect in IIH was not confirmed for erenumab, fremanezumab or galcanezumab in a retrospective chart review, at present only available in abstract form [137].

Nine out of 10 patients suffering from *trigeminal neuralgia* reported improvement in pain intensity, attack frequency and mood after erenumab treatment for 6 months in an open study [138], but this was not confirmed in a randomized placebo-controlled trial of 80 patients [139].

Erenumab or galcanezumab were also effective on migraine with aura in two patients with a *mitochondriopathy* [140, 141] and galcanezumab was found effective in a patient with *nummular headache* [142].

In a retrospective chart survey, anti-CGRP/rec mAbs markedly decreased comorbid *neuropathic pain* of various etiologies in patients treated for CM [143].

To sum up, episodic CH is at present the only headache type other than migraine where an anti-CGRP/rec mAb, galcanezumab, has evidence-based efficacy from a RCT, although the effect size is clearly smaller than in migraine. In various other headache conditions, results are discordant and mostly based on case series. In trigeminal neuralgia a RCT with erenumab was negative. Encouraging results were obtained in open studies of idiopathic intracranial hypertension, but need to be confirmed in a RCT. Such a placebo-controlled trial targeting patients with a CM phenotype would also be crucial in persistent PTH, a frequent and disabling condition in need of better management.

## What are the potential contraindications of anti-CGRP/rec mAbs?

CGRP has a widespread distribution in the human body and is involved in multiple physiological functions [5, 6]. In theory, blocking CGRP or its receptor could have various unwanted effects in the short and particularly in the long term [144, 145]. Tolerance and safety, however, were outstanding in RCTs and real-world studies of anti-CGRP/rec mAbs in migraine, even after > 6 years of administration for erenumab. Of note is that according to the Summary of Product Characteristics in most countries the only official contraindication to all 4 anti-CGRP/rec mAbs is ‘Hypersensitivity to the active substance or to any of the excipients’. Safety could be overestimated because subjects with cardio- or cerebrovascular disorders or other serious diseases were excluded from most studies [15, 146]. It must also be kept in mind that the role of CGRP or its receptor can be taken over by other neuropeptides or neuropeptide receptors and one cannot exclude that some adverse effects may take longer to appear with continuing anti-CGRP/rec mAb therapy [144, 145].

Table 3 is a synoptic overview of major physiological roles of CGRP (1st column), disorders in which CGRP blockade could have potential deleterious consequences (2nd column) and available experimental and particularly clinical evidence for their occurrence (3rd column). The 4th column shows the respective recommendations that we propose for clinical practice on the basis of available data: anti-CGRP/rec mAbs would be “contraindicated” if there is clinical and/or strong experimental evidence for a harmful effect with serious consequences; they would be “not recommended” if there is circumstantial evidence for a possible worsening effect without proven serious consequences (precautionary principle); they would need “surveillance” if the contraindications are only theoretical or based on single case reports. We will comment on some of them.

**Table 3:** Potential contraindications of anti-CGRP/rec mAbs based on physiological data & clinical evidence

Physiological CGRP functions	Potential contraindications	Experimental / clinical evidence	*RECOMMENDATION*
**Protective vasodilatation and proangiogenic effect** (reviews: 5,6) **Antioxydant and homeostatic properties** (reviews: 7,8)	Ischemic stroke	Gepants worsen ischemic cerebral outcome in mice [147] No effect on vasodilatory / contractile responses of human cerebral arteries (erenumab) [148] 1 case with cerebral proliferative angiopathy (erenumab) [152] 1 case with reversible cerebral vasospasm (erenumab + triptan + contraceptive pill) [153]	***Contraindicated ****if recent stroke* *Not recommended* *if history of stroke (precautionary principle)*
	Coronary artery disease	No worsening of angina (1 erenumab dose) [149] Vascular safety profile in RCTs similar to placebo (erenumab) [150] No case reports [151]	***Contraindicated ****if recent myocardial infarction or unstable angina* *Not recommended* *if history of myocardial infarction (precautionary principle)*
	Raynaud’s	5,3% with microvascular complications (5 cases erenumab, 3 galcanezumab, 1 fremanezumab) (2 with sclerodermia) [157] More prevalent than with triptans or beta-blockers in WHO VigiBase® [156]	***Contraindicated ****if severe Raynaud’s with microvascular lesions and/or sclerodermia*
	Arterial hypertension	62 cases (erenumab) reported to FDA in 2 years (31% had pre-existing hypertension) [160] Incidence not different from placebo in RCTs (erenumab) [159] Significant overall rise in blood pressure, *de novo* hypertension in 4/109 (3.7%) patients on erenumab, 0/87 with fremanezumab [161] Blood pressure worsening in 23.3% of 335 patients with erenumab [162]	*Surveillance of blood pressure* *Erenumab not recommended* *in severe and/or uncontrolled hypertension*
	Peripheral artery disease	No case reports [151]	*Not recommended* *if severe (precautionary principle)*
	Venous thrombosis/embolism	No case reports [151]	*Not recommended* *if recent (precautionary principle)*
	Reversible cerebral vasoconstriction syndrome	1 case report (erenumab) [163]	*Not recommended (precautionary principle)*
	Erectile dysfunction	1 case report (galcanezumab) [164]	*Surveillance*
**Vasoregulation of utero-placental blood flow & feto-placental development ***(163)* **Penetration in milk during lactation ***(143)*	Pregnancy Lactation	No deleterious effect in monkeys (erenumab) [166] Fetal mortality increase and growth decrease with s.c. infusion of CGRP_8 − 37_ (a CGRP receptor antagonist) in pregnant rats [167] No deleterious effect in case reports (erenumab) [168] and 92 safety reports [169] No signal in WHO pharmacovigilance database (VigiBase^®^) [170]	***Contraindicated ****(precautionary principle)*
**Regulation of gastro-intestinal tract motility & mucosal integrity** *(169)*	GI motility disorders	*Calcrl* gene expression 5x higher in enteric neurons than in vascular cells/sensory neurons [173] 17% of 24,573 adverse effects related to GI disorders In FAERS 2019 in [174] Constipation prevalent (20%) with erenumab, but discontinuation rare [15] CGRP causes gastrointestinal hyperactivity [172] and increases Glucagon-like Peptide-1 (GLP-1) [174]	*Erenumab not recommended* *if severe constipation* *Surveillance in Irritable Bowel Syndrome*
	Peptic ulcer, inflammatory bowel disease	No signal in databases	*Surveillance in Inflammatory Bowel Disease*
**Proliferation of keratinocytes, VEGF upregulation & reduction of inflammatory mediators** *(143)*	Cutaneous lesions	Impaired wound healing (erenumab-1 case) [176] Severe ecchymosis (erenumab + fish oil) in 1 case *(177)* Alopecia: women, non-serious, case reports and signal in FAERS (all 4 mAbs) [178–180]	*Not recommended* *if severe skin lesions or poor wound healing (precautionary principle)* *Surveillance in vasculitis & bleeding disorders* *Not recommended* *in women with abnormal hair loss*

A possible deleterious consequence of blocking CGRP’s role in protective vasodilatation, pro-angiogenesis, oxidative stress and homeostasis [5–8] has been an early concern, because it could potentially aggravate *vascular disorders and ischemia* [146]. Small molecule CGRP receptor antagonists (so-called gepants) may worsen ischemic cerebral outcome in mice following middle cerebral artery occlusion, due to dysfunction of collateral circulation [147]. A comparable study is not available for anti-CGRP/rec mAbs. However, erenumab had no effect on vasodilatory or contractile responses of human isolated cerebral arteries in vitro [148]. A single intravenous administration of erenumab 140 mg in patients with stable angina did not aggravate exercice-induced angina or ST-segment depression [149], but this study was criticized because it explored a single acute administration too short before the treadmill test to allow for complete tissue distribution and comprised few women with angina in whom the distal coronary artery system, the most sensitive to CGRP, is chiefly involved. With a follow-up now exceeding 6 years, serious treatment-related vascular adverse events have not been reported with anti-CGRP/rec mAbs [150, 151], with the exception of 2 case reports complicated by comorbid features: an ischemic stroke in a patient with cerebral proliferative angiopathy [152], and reversible cerebral vasospasm in a patient treated with erenumab, a triptan and a combined contraceptive pill [153]. Despite the absence up to now of a signal of worse outcomes for cerebral or coronary ischemia under anti-CGRP/rec mAbs, their use is contraindicated in patients with recent stroke, unstable angina or myocardial infarction. There is an expert consensus that they are also contraindicated in patients with a history of stroke or myocardial infarction [154], but this may be regarded merely as a precautionary principle that might not necessarily apply to individual disabled patients in need of an effective preventive treatment.

*Raynaud’s* phenomenon is since a long time known to be more prevalent in patients with migraine [155]. Its incidence with anti-CGRP/rec mAbs is higher in the WHO Vigibase^®^ [156] than with triptans or beta-blockers and severe Raynaud’s with microvascular complications was reported in 9 patients among whom 2 had sclerodermia [157]. It is worth mentioning that new onset digital Raynaud’s was also reported in 2 patients taking a small molecule CGRP receptor antagonist [158].

Although *arterial hypertension* did not occur more frequently in RCTs of erenumab [83, 159], 62 cases were reported to the FDA after real-world use, 31% of whom had preexisting hypertension [160]. In a Dutch study, next to a significant overall rise in blood pressure, *de novo* hypertension appeared in 3.7% (4/109) of patients treated with erenumab and in none out of 87 patients treated with fremanezumab [161]. In a recent study on the risk of hypertension after initiation of erenumab in the post-marketing setting published in abstract form, blood pressure increase occurred in 23.3% of 335 patients irrespective of pre-existing hypertension [162].

There are up to now no reports on worsening of *peripheral artery disease* or occurrence of *venous thrombosis or embolism* [150], while single case reports of *reversible cerebral vasoconstriction* with erenumab [163] and *erectile dysfunction* possibly due to galcanezumab [164] have been published.

In animal experiments, CGRP is involved in *vasoregulation of utero-placental blood flow* and feto-placental development [145, 165] and anti-CGRP/rec mAbs can penetrate in milk during lactation [166]. No deleterious effect was found in pregnant monkeys [166], but increased fetal mortality and decreased growth was found with CGRP_8 − 37_, a CGRP receptor antagonist, in pregnant rats [167]. However, no signal for an effect on the human fetus or pregnancy outcome has emerged up to now in case reports [168, 169] or in the recently updated WHO pharmacovigilance database of 286 safety reports [170]. Although based on our abovementioned criteria the term “not recommended” could be used for anti-CGRP mAbs in pregnancy, “contraindicated” was agreed upon in Table 3, because the exclusion of foeto-maternal toxicity may need much larger databases and follow-up of children after birth. Moreover, the contraindication is unlikely to be worrisome for female migraineurs, as most of them will improve during pregnancy.

*Gastrointestinal (GI) disorders* are prevalent in migraine patients and could be related to the effect of CGRP on GI tract motility and mucosal integrity [171, 172]. Though not found in RCTs, erenumab induced or aggravated constipation in real-world studies in 20% of patients on average [15]. However, constipation rarely leads to treatment discontinuation and seems to occur less frequently with the ligand-blocking mAbs. Whether these GI adverse effects are due to the high expression of the Calcrl gene, which encodes the CGRP receptor component to which erenumab binds [173], to a decrease in Glucagon-Like Peptide 1 (GLP-1) [174], to the fact that amylin binds to the CGRP receptor [171] or to their combination remains to be determined. There is no signal in available databases suggesting that the anti-CGRP/rec mAbs might worsen peptic ulcer or inflammatory bowel disease (IBD). Interestingly, however, the Fab’2 fragment of fremanezumab decreases experimental colonic hypersensitivity in rat [175], an effect that would rather be beneficial in irritable bowel syndrome.

CGRP plays a role in a number of *other organs and (patho)physiological functions.* We have previously discussed its possible effect on bones in relation to the use of anti-CGRP/rec mAbs in children [73]. Its role in skin biology may explain why the monoclonals can cause in rare patients impaired wound healing [176], ecchymosis [177] and, as reported in several case series and the FDA Adverse Event Reporting System (FAERS), alopecia [178–180]. Occurrence or aggravation of inflammatory disorders like arthritis, psoriasis and urticaria was reported in 7 patients, likely related to the role of CGRP in innate immune response and inflammation [181]. The latter could also be responsible for the recurrent oral candidiasis reported in a patient both during erenumab and galcanezumab treatment [182]. On the other hand, galcanezumab reduced osteoarthritis-related pain in rodents [183].

Although CGRP may have contrasting effects in broncho-pulmonary physiology [5], it could amplify bronchoconstriction [184] and surveillance only is recommended in patients treated with anti-CGRP/rec mAbs since no deleterious signals in obstructive pulmonary disease or in pulmonary hypertension have yet appeared in pharmacovigilance databases.

CGRP can influence the hypothalamo-pituitary axis, glucose and lipid metabolism [5, 144, 145], but up to now no hormonal dysregulation, significant weight changes or occurrence/aggravation of diabetes have been detected during anti-CGRP/rec mAb treatment.

Finally, CGRP is present in motor nerve endings at the skeletal muscle endplate where it has a trophic role [185] and increases acetylcholine release in rodents [186]. Besides occasional benign muscle spasms, chiefly at the injection site [187], adverse effects related to skeletal muscles have not been reported yet. The occurrence of a restless legs-like syndrome found in two patients treated with erenumab or galcanezumab needs to be confirmed [188], but is unlikely to be mediated by a skeletal muscle mechanism.

To summarize, because of the known physiological actions of CGRP and signals in adverse effect reporting systems and/or case reports, and chiefly as a precautionary principle, anti-CGRP/rec mAbs are *contraindicated* in subjects with recent stroke, unstable angina, myocardial infarction or severe Raynaud’s phenomenon. They are *not recommended* in uncontrolled hypertension (particularly erenumab), severe peripheral artery disease, recent venous thrombosis or embolism, severe constipation (only erenumab), severe skin lesions or poor wound healing, abnormal hair loss and severe inflammatory disorders. They are *also contraindicated* during pregnancy and breast feeding as a precautionary principle. *Surveillance* is recommended to detect aggravation of irritable bowel syndrome and inflammatory bowel disease, vasculitis and bleeding disorders, bronchopulmonary disorders, erectile dysfunction, hormonal dysfunctions and muscle disorders.

## Data Availability

Not applicable.
